# Evaluation of biomarkers in olfactory neuroepithelial cells and plasma obtained from Mexican patients with Mild Cognitive Impairment and possible Alzheimer's disease

**DOI:** 10.1002/alz70856_102307

**Published:** 2025-12-25

**Authors:** Maria‐del‐Carmen Silva‐Lucero, Obed‐Ricardo Lora‐Marin, Andres‐Ivan Gutierrez‐Malacara, Juan‐Ramon Padilla‐Mendoza, Rafael Jijon‐Lorenzo, Maria‐del‐Carmen Cardenas‐Aguayo

**Affiliations:** ^1^ UNAM, School of Medicine, Department of Physiology, CDMX, DF, Mexico; ^2^ Neurosciences Master in Science Program INB, UNAM, CDMX, DF, Mexico; ^3^ División de Químico Biológicas, Universidad Tecnológica de Tecámac, Mexico, EM, Mexico

## Abstract

**Background:**

Mild cognitive impairment is a nosological attempt to identify individuals who are at an intermediate point on the continuum between cognitive health and dementia, which may increase the risk of Alzheimer's disease (AD). AD is the most common cause of dementia; substantial neuronal loss and neuropathological lesions can damage many brain regions. Symptoms of the disease begin with mild memory difficulties and evolve towards cognitive impairment. The lack of disease‐modifying therapies combined with the long presymptomatic phase highlights the need to identify individuals early in the disease process to develop putative interventions. Novel biomarkers include PET scans, which show great promise for clinical and research use but are expensive approaches; here, we propose a more affordable approach to detect biomarkers in cells isolated from the olfactory neuroepithelium and plasma.

**Method:**

Older adults with signs of Mild Cognitive Impairment and healthy individuals of the same age were recruited. We performed a MoCA Test, a medical interview, and obtained a blood sample for plasma and an olfactory test for each volunteer. A noninvasive nasal exudate was performed to obtain, isolate, and characterize Olfactory Epithelial Precursor Cells. Once the cultures were established, different biomarkers were evaluated using the Western Blot technique.

**Result:**

A cohort of patients with cognitive impairment and healthy controls were recruited. We obtained from all volunteers their clinical history, a MoCA‐cognitive test, blood samples, and the culture of the Olfactory Neuroepithelial Precursor Cells of each patient was established. The following markers were analyzed by Western Blot Amyloid precursor protein, total tau (T‐tau) and phospho‐tau217 (*p*‐tau217), as well as the AD biomarkers selected by our bioinformatics analysis: calcineurin subunit, a serine/threonine phosphatase under the control of Ca2+/calmodulin; b‐synuclein, which is associated with synaptic degeneration; a‐synuclein. Our data showed no correlation between p/tau217and cognitive impairment. Aβ levels have been measured in plasma.

**Conclusion:**

The search for altered genes in mild cognitive impaired patient‐derived peripheral cells will allow us to identify potential early biomarkers of AD that could be validated in blood samples from patients with AD, as a possible affordable early diagnostic tool.

